# Intersectionality, health equity, and EDI: What’s the difference for health researchers?

**DOI:** 10.1186/s12939-022-01795-1

**Published:** 2022-12-19

**Authors:** Christine Kelly, Lisette Dansereau, Jennifer Sebring, Katie Aubrecht, Maggie FitzGerald, Yeonjung Lee, Allison Williams, Barbara Hamilton-Hinch

**Affiliations:** 1 Department of Community Health Sciences, University of Manitoba, Winnipeg, Manitoba Canada; 2 Department of Sociology, St. Francis Xavier University, Antigonish, Nova Scotia Canada; 3 Department of Political Studies, University of Saskatchewan, Saskatoon, Saskatchewan Canada; 4 Faculty of Social Work, University of Calgary, Calgary, Alberta Canada; 5 Chung-Ang University, Seoul, South Korea; 6 School of Earth, Environment & Society, McMaster University, Hamilton, Ontario Canada; 7 School of Health and Human Performance, Dalhousie University, Halifax, Nova Scotia Canada

**Keywords:** Intersectionality, Equity, diversity and inclusion, Health equity

## Abstract

Many countries adopted comprehensive national initiatives to promote equity in higher education with the goal of transforming the culture of research. Major health research funders are supporting this work through calls for projects that focus on equity, resulting in a proliferation of theoretical frameworks including “intersectionality,” “health equity,” and variations of equity, diversity and inclusion, or EDI. This commentary is geared at individual principal investigators and health research teams who are developing research proposals and want to consider equity issues in their research, perhaps for the first time. We present histories and definitions of three commonly used frameworks: intersectionality, health equity, and EDI. In the context of health research, intersectionality is a methodology (a combination of epistemology and techniques) that can identify the relationships among individual identities and systems of oppression; however, it should also be used internally by research teams to reflect on the production of knowledge. Health equity is a societal goal that operationalizes the social determinants of health to document and address health disparities at the population level. EDI initiatives measure and track progress within organizations or teams and are best suited to inform the infrastructure and human resourcing “behind the scenes” of a project. We encourage researchers to consider these definitions and strive to tangibly move health research towards equity both in the topics we study and in the ways we do research.

**Supplementary Information:**

The online version contains supplementary material available at 10.1186/s12939-022-01795-1.

## Introduction

The time for social justice in research and higher education, it seems, is now. Many countries, including Canada, the United Kingdom, Australia, and the United States of America, adopted comprehensive national initiatives to promote equity in higher education with the goal of “deeper cultural change within the research ecosystem” [1–4]. Research funders and universities have employees, departments, and strategic plans dedicated to *equity, diversity and inclusion* (EDI) and variations on this phrase.^1^ While there is a new sense of urgency, this moment is embedded in an ongoing history of interventions from feminist and women’s studies scholars who call upon researchers to include gender as a category of analysis [5, 6], and perhaps more difficult, for institutions and academic fields to include women as researchers and producers of knowledge [7]. There are parallel efforts addressing the inclusion of people of African/Black descent, people of color, people with disabilities, diverse gender identities, Indigenous people, and additional groups who are historically marginalized in higher education and research settings.^2^

In the context of team-based health research, competitive funding supports large scale, high impact studies and is used as a key metric for individual career progression. The transformative EDI initiatives cited above acknowledge the importance of funding in academia and seek to redress systematic biases in research award allocations. A study looking at research funding gaps at the Canadian Institutes of Health Research found gender disparities related to “less favorable assessments of women as principal investigators" [8]. Notably the authors of the study were not able to analyze based on race or other relevant characteristics due to lack of data collection by the funding agency at that time [8]. In 2011, a landmark review of the National Institute of Health Research (USA) found “applications from white Principal Investigators (PIs) were 1.7 times more likely to be funded than applications from African-American/Black PIs” [9]; in 2019, this disparity still existed [10, 11]. There is a stark funding divide between research teams in the global north compared to those in the global south [12]. Yet, despite these documented gaps in research funding, it is also recognized that diverse research teams lead to more innovative research [13] and research that is more sensitive to the needs of equity-seeking communities [14].

In tandem with growing recognition of systematic bias in funding allocations, major health research funders call for research that focuses on equity topics [15–19]. The growing interest in equity-oriented projects is laudable, but also leads to an influx of established researchers tackling the topic for the first time. Researchers describe a phenomenon dubbed “health equity tourism” [20], where (white) high impact researchers from other fields successfully apply to health equity funding opportunities building on their previous funding track record, producing research with easily avoidable mistakes. As Lett et al. explain, studying issues such as structural racism requires a careful, nuanced approach and collaboration with people with lived experience, and health equity “tourists” can incorrectly assume that their conventional research methods are transferable to this context [21]. The resulting studies fail to capture the complexity of racial disparities, do not produce meaningful or actionable results, and may even risk perpetuating racial stereotypes and biases. Similarly, Smith et al. 2018 argue health equity is too often divorced from social justice aims and focuses more on “proximal” disparities rather than structural drivers [22]. Some scholars argue that institutions and individuals tend to undertake EDI work in a performative manner, without actually confronting systemic causes of inequity and exclusion within their structures [23, 24].

In response to the growing demand for equity research balanced with the problem of health equity tourism, this commentary is geared at principal investigators and health research teams who are developing research proposals and want to consider equity issues in their research, perhaps for the first time. In particular, we distinguish three commonly used concepts: intersectionality, health equity, and EDI. We offer guidance for interdisciplinary health research teams at the conceptualization stage of their research projects and when deciding among these approaches. We present the history and definitions of each term and summarize key differences, similarities, and considerations for use. In the context of research, intersectionality is a methodology (a combination of epistemology and techniques) that can identify the relationships among individual identities and systems of oppression. Intersectionality typically includes an explicit commitment to social justice and is a common paradigm mobilized in grassroots activist settings. Health equity is a societal goal. As a research framework, health equity operationalizes the social determinants of health to document and address health disparities at the population level. EDI initiatives measure and track progress towards “diversity” within organizations or teams and are best suited to inform the infrastructure and human resourcing “behind the scenes” of a project. We encourage researchers to consider these definitions and strive to tangibly move health research towards equity both in the topics we study and in the ways we do research.

### Defining intersectionality

Intersectionality posits that individual identities and social locations such as gender, race, and class intersect and reflect systems of oppression such as sexism and racism [25]. Intersectionality is attributed to critical race theorist and feminist legal scholar Kimberlé Crenshaw [25] and the activism of the Black, feminist, and lesbian Combahee River Collective (1977). In the late 1970s, these and other Black feminist activists and scholars, were excluded from *both* the women’s movement and the anti-racist movement [26, 27]. It is integral to reference this history when using intersectionality as the contributions of Black women scholars are often erased through abstraction. The overlap of multiple identities, or *intersections,* represent unique experiences that are overlooked by focusing on one identity over another [28]. As such, intersectionality helps explore differences within and among groups. Intersectionality typically includes an explicit commitment to social justice—that is, an aim to redistribute wealth, opportunities, and privileges at a societal level [29]. Achieving social justice would require a dramatic re-orientation of contemporary institutions, laws, and economic systems.

Intersectionality suggests that privilege and oppression shift based on context, and thus one may be privileged in one context but disadvantaged in another. For example, while all women may be subject to discrimination based on gender, Black women have distinct experiences of sexism and racism. Intersectionality is a successful theoretical and activist intervention. It is a core orientation in women’s and gender studies, remains commonly used as a paradigm in activist groups, is cited as a theoretical approach in many empirical studies [30–32], and is a focal point for high-level theorizing [33, 34]. It is so successful that there are concerns that it may be a “buzzword” [35, 36].

In the context of health research, intersectionality shares affinities with other ideas from feminist methodologies and these perspectives are often gathered through qualitative research. For example, standpoint epistemology argues that people in the margins have clearer knowledge about structures of oppression than those at the centers, and so foregrounds marginalized voices [26, 37]. Intersectionality requires researchers to be *reflexive* of their own social locations and state a theoretical orientation (rather than attempt to control for bias) in protocols and publications [38]. Reflexivity applies to the entire research process, including the formation of the team, hiring, and recruitment of participants. There are also efforts to develop intersectionality measures to be used in survey research [39–42]. However, intersectionality loses its historical connection when used only as a method in data collection/analysis rather than as a comprehensive *methodology* incorporating reflexivity on behind the scenes research processes [43]. That is, teams “using” intersectionality in their research must also “do” intersectionality through practices such as self-identification questionnaires to ensure diversity on the team, providing additional mentorship or opportunities to students and scholars who may face barriers in academia, and cultivating an awareness of bias and discrimination that can be perpetuated through research [44].

### Defining health equity

Health equity is a societal goal of global and public health research and practice, seeking to eliminate unjust health disparities at the population level that are shaped by the social determinants of health [45]. The World Health Organization (WHO) describes: “Health equity is achieved when everyone can attain their full potential for health and well-being” [46]. Historical research traces the concept of health equity back as far as 1801, and the 1948 constitution of the WHO formally endorsed elements of the contemporary concept [47]. Health equity gained momentum and an explosion of interest in the 1990s following Marmot’s highly influential work on the social determinants of health [48–50], that is “non-medical factors that influence health outcomes” [51]. Research framed with the social determinants of health has generated a significant technical evidence base that documents health disparities within and between populations through the use and creation of local, national and international health datasets [51].

In 2008, the WHO Commission on Social Determinants of Health outlined three principles of action required to achieve health equity: i) improve the conditions of daily life, ii) tackle structural drivers of health, that is the inequitable distribution of power, money, and resources, and iii) measure and evaluate outcomes [52]. The second action resonates with the definition of social justice mobilized in intersectionality, but this area of action proves the most difficult to apply in the context of health research. Braveman recently posited a complete definition of health equity that emphasizes structural drivers and social justice:

“Health equity means that everyone has a fair and just opportunity to be as healthy as possible. This requires removing obstacles to health such as poverty, discrimination, powerlessness, and their consequences—including lack of access to good jobs with fair pay, safe environments, and quality education, housing, and health care. For the purposes of measurement, health equity means reducing and ultimately eliminating disparities in health and its determinants that adversely affect excluded or marginalized groups.” [53].

As with intersectionality, surging popularity has led to some confusion. Specifically, not all measured differences in health outcomes qualify as *health disparities,* which refers to differences in health outcomes that occur among populations who are economically and/or socially disadvantaged [54]. Populations who systematically and persistently experience disadvantage include people from lower socio-economic backgrounds, racial or ethnic populations, women, Indigenous groups, and 2SLGBTQIA+ people. In a global health context, disadvantaged populations may also refer to the entire population of a low-income country.

### Defining equity, diversity, and inclusion

Equity, diversity, and inclusion (EDI) is a policy-focused initiative aimed at addressing the ongoing exclusion of under-represented groups in employment, education, and other institutional contexts. EDI emerged out of the American Civil Rights Movement in the 1960s as a “response to deeply entrenched patterns of racial discrimination” [55]. Early iterations emphasized increasing the numbers of people from groups that have been disadvantaged in the workplace [55]. While the policy context and development of EDI varies from country to country, it is generally recognized that the terms equity and diversity replace previous discourses of ‘affirmative action’, ‘equal opportunity’ and ‘employment equity’ in human resource policies [56, 57]. Further, EDI takes up the shortcomings of previous policies by moving beyond numerical representation to fostering meaningful, sustainable change toward inclusion in the workplace, education, and broader public sphere.

It is important to distinguish equity from equality. Equality refers to treating all people the same (e.g., offering the same opportunities), while equity refers to achieving fair outcomes, recognizing diversity, and addressing inequality through intervention [24]. Diversity refers to the welcoming and embracing of difference, in relation to social demographics as well as a diversity of perspectives and ideas [24, 58, 59]. Inclusion is a critical component to EDI, representing the idea that it is not enough to invite a variety of people into institutions [23]. Inclusion is fostering an environment and culture that is welcoming and supports diverse individuals and/or groups of people, and may also require concrete changes (e.g., accommodations to address physical and social barriers to inclusion) [59].

There are other variations of the EDI acronym that bring their own histories and perspectives, most commonly the addition of “A” for access or accessibility, drawing on the history of disability rights and accessibility policies and legislation. There is sometimes a “D” to bring in the complex and unique experience of Indigenous people working towards decolonialization; this is more common in Canada and Australia than other countries with smaller Indigenous populations.

While EDI efforts typically focus on institutional level change, funding bodies are increasingly requiring researchers and research teams to implement EDI within their research designs (e.g., participants, budgeting lines for accessibility) and in research practices in a way that echoes feminist reflexivity. For example, the Canadian funding body, the Social Sciences and Humanities Research Council produced a guide to implementing EDI in research practice and research design [60]. EDI in research practice refers to “promoting diversity in team composition and trainee recruitment” and “fostering an equitable, inclusive and accessible research work environment” while EDI in research design might involve using “intersectionality, gender-based analysis plus, anti-racist approaches, and disaggregated data collection and analysis that includes consideration of diversity and identity factors” [60]. EDI can be used as an institutional mechanism for compelling health researchers to consider the exclusions of specific teams and projects.

## Discussion: considerations for using each concept

Our brief historical explanations and definitions show that these three concepts overlap. The researchers, theorists, and activists were likely informed by each other’s contributions. Most notably, all three have an awareness of injustice among and within groups. Table 1 outlines the background of each concept, and their respective application to the health research context including the strengths and limitations of each concept. Further, we offer external resources for the application of each concept in Additional file 1: Appendix A.

**Table 1:** Summary Intersectionality, health equity and EDI

	Intersectionality	Health equity	EDI
Disciplinary background	Legal Studies Women and gender studies Used broadly in many social sciences and humanities, and more recently in some health fields	Global Health Public Health Population Health Social Medicine	Education and employment policy
History	Black feminist theorists and activists in the late 1970s in the United States	Longstanding roots, conceptually part of WHO founding constitution (1948) Well-established in health research	Affirmative action and civil rights movements (US); Employment equity (Canada) Race Relations Act (UK)
Level(s) of analysis	Individual and systems of oppression	Populations and health systems	Institutions or teams
Strengths	• Ideal for exploring complex social locations with multiple factors at play • Helps to transform higher education by valuing lived experience on the research team and through research • Aligns with many qualitative research methods • Encourages reflexivity among researchers	• Resonates for those in health sciences and other disciplinary backgrounds • Exceptional for comparing populations within or across countries • Pairs well with epidemiology, quantitative, and longitudinal approaches • Supplies ethical grounding for health services research	• Measurement – concreteness • Completeness – use in research practice *and* development • Can be used for research teams with less emphasis on social theory • Can be linked to training requirements
Limitations	• Too conceptual – difficult to apply in concrete ways in research contexts • Difficult to apply in quantitative research, although progress is being made in the development of intersectionality measures	• Does not require reflexivity on the production of knowledge/norms of academia/construction of the team • Focus on health outcomes may risk ignoring, minimizing, or causing other outcomes and unintended consequences • Limitations arise when structural drivers are not considered	• Cannot be used as a research methodology or theoretical framework • Difficult to address structural/systemic barriers • Requires considerable time and labour to implement effectively • In most forms, can add additional service burdens to faculty and students who have been marginalized • Risks a “check list” approach through quotas

### Considerations for intersectionality

There are three key considerations when using intersectionality in team-based health research context. First, it is important to assess team members’ level of comfort with committing to social justice or an explicitly feminist approach. Some scholars argue that research using intersectionality must openly strive towards social justice [33], while others argue that when people “use” intersectionality as a framework it will inadvertently achieve the same ends [61, 62]. Secondly, it is important to consider that intersectionality is the most coherent when applied as a methodology; that is, an approach that informs the composition of the research team, formation of the question, approach to recruitment, *and* a method used in data collection and analysis. Finally, with the exception of intersectionality measurement work, a common criticism is the difficulty in applying an intersectional framework in a concrete way. It can be challenging to: determine which intersections are relevant to a particular topic (as it is not possible to explore *all* intersections); whether gender must always be considered, and; how to approach Indigenous perspectives, some of whom do not want colonial legacies subsumed as one of many identities on an extensive list [63]. There are guides on how to apply intersectionality and a notable application is the gender-based analysis plus framework endorsed by the Canadian government. Overall, there are limited resources for a research context, and even fewer for studies using a quantitative or mixed-methods approach.

### Considerations for health equity

Striving for health equity requires using a social determinants of health framework, which are widely established with numerous tools and models---so many that it can be overwhelming to choose a one for a given health research topic [64]. Nevertheless, the framework must be chosen with care as some fail to embrace consideration of the structural drivers of health and lack the ontological foundations to help understand the social complexity [65, 66]; some scholars in fact call for the incorporation of intersectionality to address this gap [40, 67].

Social justice is a fundamental principal of health equity, yet some research has tried to hold the two ideas as conceptually distinct, perhaps framing justice-based considerations as political and lacking in objectivity [68]. Braveman comments, “‘Equity’ means justice: and justice is often a contentious issue.” Only one-third of existing health equity frameworks help to identify causal factors while perhaps even fewer recognize that there are “causes of the causes” or “root causes” [66]. This is a weakness in the application of healthy equity rather than the conceptualization, as numerous authors and key reports emphasize the importance of structural drivers and a justice orientation. The core mandate for social transformation can be misapplied when researchers lack training in critical theories and anti-oppressive practice, and when health disparities are rooted in complex social phenomenon such as violence and poverty. The focus on populations rather than individual locations can erase complexity of those who occupy multiply-marginalized positions. Researchers should also pay attention to how health equity is understood in a given context and among the population(s) being studied.

### Considerations for EDI

EDI is neither a methodology like intersectionality or a societal goal like health equity; yet is is a valuable organization tool for assessing and implementing change. When designing EDI policies, a critical consideration is to develop mechanisms of measurement and accountability. A concern of many EDI practitioners is that institutions may be appearing to ‘do EDI’ by having an employee, officer, or policy on the matter while not making any changes to the norms and practices that make it difficult to thrive in the institution or industry in the first place [23, 24]. To avoid this, EDI policies must be accompanied with concrete plans for tracking progress and a means of holding the members of an institution accountable to the policy.

Critiques of EDI have been raised that the softening of language from anti-racism to equity, diversity and inclusion masks the social justice origins of EDI [56]. As such, EDI plans must be explicit in their aims and approach EDI as an ongoing process of assessment, critical reflection, and revisions as needed. To make meaningful change with EDI, structural and systemic barriers must be considered and accounted for. In a research context, a major strength of EDI is that it can be applied to internal research practices of quantitative and mixed method research teams, without it having to be the theoretical framework of a study.

Finally, it is important to collaborate with members of groups that have been under-represented when developing EDI plans to ensure they meet the needs of the communities they aim to serve. It is equally important that individuals who have been multiply marginalized do not get saddled with the majority of EDI work. This is a common experience faced by women of colour and Black women in the academy who undertake significant often-invisible labour of advancing EDI, while their white colleagues continue to advance their research, thereby perpetuating the very inequities EDI means to address.

## Conclusion

Intersectionality, health equity, and EDI can be used in tandem or independently, yet they are not interchangeable. In the context of health research, intersectionality is a methodology for identifying and understanding the relationships between identity and systems of oppression while health equity is a goal that calls us to focus on inequities at the population level; EDI emphasizes measuring progress and metrics to concretely demonstrate how an institution or team is moving towards equity, diversity, and inclusion.

An essential component of using each concept is ensuring that the research team (or organization) includes individuals who are well-versed in the nuances of each concept. This will help mitigate the risk of becoming a “buzzword” or heath equity tourism. Institutional EDI training may be an avenue to helping health researchers distinguish these frameworks, and the educational aspect of EDI is a core strength. In the case of intersectionality, this means including women’s and gender studies scholars, feminist, or critical race theorists, and significantly, people who are racialized and socially located in ways that result in lived experiences of marginalization. It is easier said than done in health research, as the disciplinary training and practices differ substantially and because of the historical exclusion of variously located people within health research. Health equity requires the consultation of public health researchers, or other researchers with an appropriate understanding of the social determinants of health, to ensure that the relevant structural drivers are considered. In implementing EDI plans or policies, it is vital the work be led by individuals with lived experience of systemic oppression as well as experienced EDI practitioners, while being careful not to over-burden individuals from groups that have been marginalized.

If the time for institutional transformation in health research is now, health researchers have plenty of tools at the ready to advance equity, diversity, and inclusion in all aspects of academic work. While these concepts can be complementary, they are not interchangeable, and it is important to choose with intention to ensure the maximum effect of these frameworks.

## Supplementary Information

**Additional file 1.**

## Data Availability

Not applicable.

## Footnotes

1 There are variations of this acronym, including Diversity, Equity and Inclusion (DEI). There are versions that highlight accessibility for people with disabilities, such as Inclusion, Diversity, Equity and Access (IDEA) or Equity, Diversity, Inclusion and Accessibility (EDIA). Some places work from a decolonial lens to address the injustices faced by Indigenous people, for example Equity, Diversity, Inclusion and Decolonization (EDID). In this brief paper, we use EDI for simplicity.

2 E.g., Black Health Education Collaborative (https://www.bhec.ca); Black Medical Students’ Association of Canada (https://www.bmsac.ca/); Coalition of Disability Access in Health Sciences Education (https://www.hsmcoalition.org); Researching for LGBTQ Health (http://lgbtqhealth.ca/), and Well Living House (http://www.welllivinghouse.com/)
