# Supplementary material for: Intersectionality, health equity, and EDI: What’s the difference for health researchers?
Source: Int J Equity Health. 2022 Dec 19;21:182. doi: 10.1186/s12939-022-01795-1 (PMC9764702; doi:10.1186/s12939-022-01795-1)
Supplement: Supplementary file 1 — Additional file 1. [file 12939_2022_1795_MOESM1_ESM.docx]

Intersectionality, health equity, and EDI: What’s the difference for health researchers?

**Appendix B: List of Resources**

**Health Equity**

- Health Equity Impact Assessment
  - Government of Ontario Health Equity Impact Assessment (1)
  - Health Equity Impact Assessment Workbook (2)
- Health Equity Tools 2.0 (3)

**Intersectionality**

- Everyone Belongs: A Toolkit For Applying Intersectionality (4,5)
- Feminist Intersectionality and GBA+ (6)
- Gender Based Analysis Plus Training (GBA+) (7,8)
- Intersectionality – Based Policy Analysis Framework (IBPA) (9)
- Intersectionality and Knowledge Translation Tools (10)
- Operationalizing Intersectionality Framework: A Tool for Sport Administrators (11)
- New Intersectionality Assessment Framework (12)

**Equity, Diversity, and Inclusion**

- Guide to Equity, Diversity and Inclusion in Research (13)
- EDI in Higher Education and Research (14)
- EDI Resource Bank (15)

**References**

1. Government of Ontario M of H and L-TC. Health Equity Impact Assessment (HEIA) - Ministry Programs - Health Care Professionals - MOH [Internet]. Government of Ontario, Ministry of Health and Long-Term Care; [cited 2021 Nov 30]. Available from: https://www.health.gov.on.ca/en/pro/programs/heia/tool.aspx

2. Health Equity Impact Assessment (HEIA) Workbook. Available from: https://www.health.gov.on.ca/en/pro/programs/heia/docs/workbook.pdf

3. Health Equity Tools 2.0 | National Collaborating Centre for Determinants of Health [Internet]. [cited 2022 Jan 18]. Available from: https://nccdh.ca/resources/entry/health-equity-tools-2.0

4. Everyone belongs: a toolkit for applying intersectionality | Canadian Women’s Health Network [Internet]. [cited 2021 Nov 30]. Available from: https://cwhn.ca/en/node/43489

5. Everyone belongs: a toolkit for applying intersectionality [Internet]. CRIAW. [cited 2021 Nov 30]. Available from: https://www.criaw-icref.ca/publications/everyone-belongs-a-toolkit-for-applying-intersectionality/

6. Feminist intersectionality and GBA+ - CRIAW [Internet]. [cited 2021 Nov 30]. Available from: https://www.criaw-icref.ca/our-work/feminist-intersectionality-and-gba/

7. Cameron A, Tedds LM. Gender-Based Analysis Plus (GBA+) and intersectionality: overview, an enhanced framework, and a British Columbia case study [Internet]. Rochester, NY: Social Science Research Network; 2020 Dec [cited 2021 Oct 17]. Report No.: ID 3781905. Available from: https://papers.ssrn.com/abstract=3781905

8. Gender-Based Analysis Plus (GBA+) [Internet]. [cited 2021 Nov 30]. Available from: https://www.sac-isc.gc.ca/eng/1562156595425/1562156619340

9. Hankivsky O, Grace D, Hunting G, Giesbrecht M, Fridkin A, Rudrum S, et al. An intersectionality-based policy analysis framework: critical reflections on a methodology for advancing equity. Int J Equity Health. 2014 Dec 10;13(1):119.

10. Intersectionality & KT | Knowledge Translation Program [Internet]. [cited 2021 Nov 30]. Available from: https://knowledgetranslation.net/portfolios/intersectionality-and-kt/

11. The Operationalizing Intersectionality Framework: a tool for sport administrators | Blog | SIRC [Internet]. [cited 2021 Nov 30]. Available from: https://sirc.ca/blog/operationalizing-intersectionality-framework/

12. New Intersectionality Assessment Framework [Internet]. [cited 2021 Nov 30]. Available from: https://www.ifes.org/news/new-intersectionality-assessment-framework

13. Government of Canada SS, Humanities Research Council of Canada. Guide to addressing equity, diversity and inclusion in Partnership Grant applications [Internet]. [cited 2021 Nov 1]. Available from: https://www.sshrc-crsh.gc.ca/funding-financement/apply-demande/guides/partnership_edi_guide-partenariats_guide_edi-eng.aspx#appendix-a

14. EDI in higher education and research resources - CFSG [Internet]. Chaire pour les femmes en sciences et en génie au Québec. [cited 2022 Jan 18]. Available from: http://cfsg.espaceweb.usherbrooke.ca/edi-in-higher-education-and-research/

15. EDI Resource Bank [Internet]. [cited 2022 Jan 18]. Available from: https://www.edi-resourcebank.co.uk/
